# Gastrohepatic Fistula Secondary to Metastatic Colorectal Adenocarcinoma in a Patient With Lynch Syndrome

**DOI:** 10.1016/j.gastha.2025.100807

**Published:** 2025-09-14

**Authors:** Abdulla Alabed, Faisal Abubaker, Ahmed Daibes, Sarah Altamimi

**Affiliations:** 1 Internal Medicine Department, King Hamad University Hospital, Busaiteen, Kingdom of Bahrain; 2 Gastroenterology and Hepatology, SBIM, Saudi Board of Adult Gastroenterology, Endoscopy Department, King Hamad University Hospital, Busaiteen, Kingdom of Bahrain; 3 Radiology Department, Dammam Medical Complex, Dammam, Kingdom of Saudi Arabia

**Keywords:** Gastrohepatic Fistula, Colorectal Adenocarcinoma, Gastrointestinal Bleeding

## Abstract

A gastrohepatic fistula is an abnormal connection between the stomach and the liver. It can occur in the setting of colorectal liver metastases with direct invasion into an adjacent organ, causing upper gastrointestinal bleeding. Early recognition and a multidisciplinary approach remain crucial even in advanced cases when curative options are limited; palliative care remains appropriate to guide management.

## Introduction

Colorectal cancer (CRC) is the third most common cancer worldwide and accounts for 10% of all cancers, the second most common cancer diagnosed in women, and the third in men.^1^ The liver is the most common site of metastatic disease in patients with CRC, with at least 25% developing colorectal liver metastases (CRLM) during their illness.^2^ Development of a gastrohepatic (hepatogastric) fistula secondary to CRLM is exceptionally rare. As the liver metastases erode into the stomach wall, forming the fistula, patients may present with gastrointestinal (GI) bleeding, an extremely challenging and unique presentation in clinical practice.^3^ Lynch syndrome, also known as hereditary nonpolyposis CRC, represents an autosomal dominant predisposition to CRC, endometrial carcinoma, and other cancers because of defective DNA mismatch repair.^4^ We report a case of gastrohepatic fistula secondary to metastatic liver lesions from mucinous colon adenocarcinoma in a patient with Lynch syndrome. This case not only shows the presentation of a gastrohepatic fistula with upper GI bleeding but also the aggressive clinical course of such tumors despite multimodal treatment.

## Case Presentation

A 60-year-old male, known case of multifocal right colon adenocarcinoma, moderately differentiated, pT2(m) N2b M1a (liver), diagnosed on October 21, 2021, with subsequent genetic testing confirming Lynch syndrome.

The patient underwent a laparoscopic total colectomy on January 2, 2022. He subsequently received 6 cycles of FOLFOX-Bevacizumab every 2 weeks between February 28, 2022, and May 12, 2022. A positron emission tomography - computed tomography (PET-CT) performed on June 12, 2022, demonstrated a partial response. The patient was then lost to follow-up and underwent a robotic left lateral segmentectomy of the liver lesion on October 5, 2022, abroad while under the care of a private physician. Treatment was resumed with 6 cycles of adjuvant FOLFOX-Bevacizumab every 2 weeks between November 2022 and January 23, 2023. The patient was started on Pembrolizumab, receiving 14 cycles from March 2023 to January 2024 for stage M1 disease, with no evidence of disease status. Follow-up colonoscopy in August 2023 was normal. PET-MRI in September 2023 showed no liver lesions, but a mild increase in metabolic activity and wall thickening at the ileorectal anastomosis site. PET-CT in March 2024 showed no evidence of disease. However, PET-CT in October 2024 revealed multiple hepatic focal lesions involving the left hepatic lobe, along with a newly developed left bladder wall mass. The patient was evaluated in 2024 for the bladder mass, which showed low grade noninvasive papillary urothelial carcinoma of the bladder. He received 5 cycles of Pembrolizumab between November 2024 and February 2025. A PET-CT was planned for January 2025, but the patient delayed the appointment.

On March 11, 2024, the patient presented to the Emergency Department at King Hamad University Hospital with one episode of coffee ground vomitus estimated at approximately 300 mL, on a background of abdominal pain and vomiting, primarily food content, over the preceding 10 days, along with reduced appetite and right upper quadrant pain.

On physical examination, the patient was hemodynamically stable with a blood pressure of 128/79 mmHg and a heart rate of 92 bpm. His Eastern Cooperative Oncology Group Performance Status was 2. The abdomen was soft and lax, and digital rectal examination revealed melena.

Laboratory investigations revealed anemia with hemoglobin of 7.9 g/dL, abnormal liver function tests: total bilirubin 30.5 μmol/L, direct bilirubin 18.3 μmol/L, alkaline phosphatase 841.5 U/L, alanine aminotransferase 81.0 U/L, gamma-glutamyl transferase 241.2 U/L, and aspartate aminotransferase 86 U/L. Abdominal and chest radiographs showed no free air under the diaphragm. The patient was kept nil per os. He was administered intravenous omeprazole. He was supported with 2 units of packed red blood cells and intravenous fluids (normal saline).

**Figure 1:**
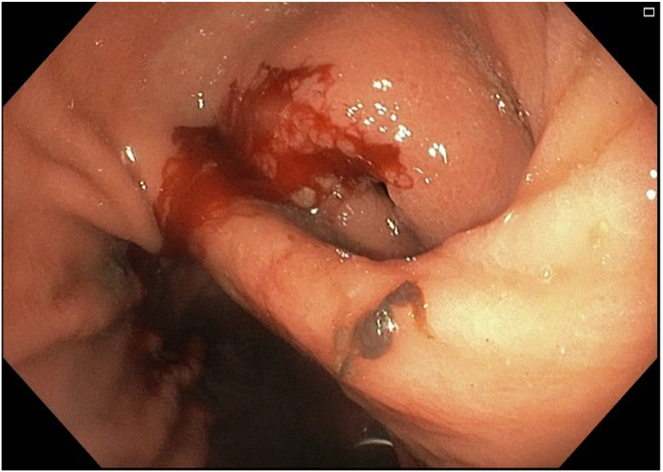
Gastroscopy findings demonstrate an actively bleeding gastric ulcerative lesion, representing the gastric opening of a gastrohepatic fistula.

**Figure 2:**
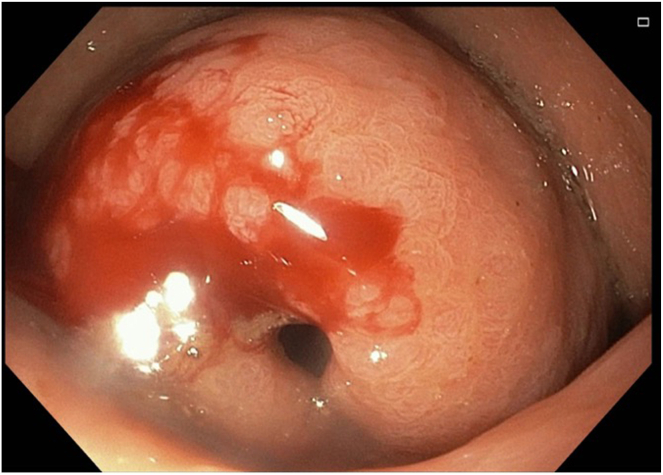
Gastroscopy findings demonstrate an actively bleeding gastric ulcerative lesion, representing the gastric opening of a gastrohepatic fistula.

**Figure 3:**
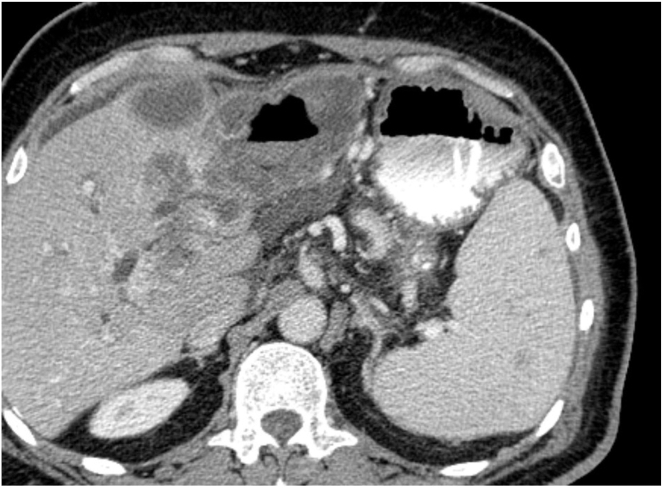
Axial CT sections with oral contrast shows a large cavitary hepatic lesion with positive oral contrast—air level with hyperdense fluid, consistent with fistulization into the gastric antrum.

**Figure 4:**
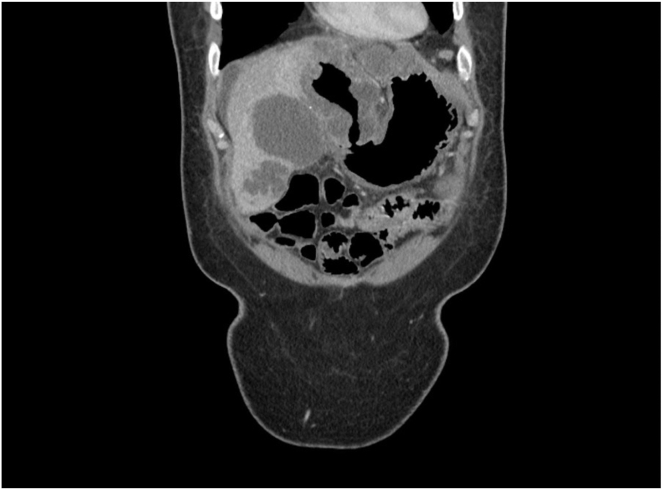
Coronal CT sections demonstrate a necrotic left hepatic mass with fistulous communication with the gastric antrum.

**Figure 5:**
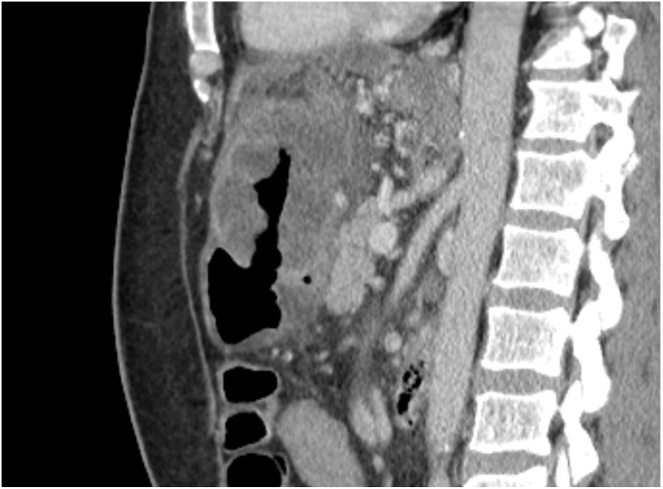
Sagittal CT sections demonstrate a necrotic left hepatic mass with fistulous communication with the gastric antrum.

The following day, the patient underwent upper GI endoscopy. White to yellow plaques were observed in the esophagus. In the stomach, the antrum appeared deformed with severe inflammation and an overlying raised lesion along the greater curvature. This lesion appeared to be caused by external compression invading the gastric wall, and it had a central opening suggestive of a possible perforation (see Figures 1 and 2). CT abdomen with contrast findings were consistent with progressive metastatic colorectal carcinoma to the liver complicated by portal vein thrombosis and large necrotic cavitary left lobe mass with gastrohepatic fistula formation (see Figure 3, Figure 4, Figure 5).

The patient reviewed by the general surgery and hepatobiliary teams. Surgical intervention was deemed unfeasible due to the extensive hepatic disease. It was advised that if bleeding were to persist, a CT angiogram followed by angioembolization would be the only viable option. He was managed conservatively, reviewed by a dietitian, and initiated on total parenteral nutrition (TPN). Intravenous omeprazole was continued. The patient's hemoglobin remained stable. By day 5, TPN was gradually tapered, and the patient was started on clear liquids. His diet was advanced to puree-like consistency, and by day 15, he was tolerating a soft diet, allowing for TPN discontinuation. On day 16, the patient was initiated on FOLFIRI (Folinic acid, Fluorouracil, and irinotecan) chemotherapy (March 26, 2025). As of April 30, 2025, he had completed Cycle 3. He was tolerating oral intake well, reported improved right upper quadrant pain, and a CT scan of the abdomen and pelvis was planned after completion of Cycle 6.

## Discussion

A “fistula” is an abnormal connection between 2 epithelial surfaces. Fistulae are named based on the 2 surfaces or lumens they connect to 5. A gastrohepatic fistula is formed between the liver and the stomach. It has been reported in the literature as a result of infections such as liver abscess,^6^ malignancies such as hepatocellular carcinoma,^7^ and rupture of a pseudoaneurysm.^8^ The presentation of upper GI bleeding in gastrohepatic fistula has also been reported.^9^ In the setting of CRLM, to our knowledge, gastrohepatic fistula has been reported as a postprocedure or treatment complication in 2 cases^10^^,^^11^; however, spontaneous or direct invasion into adjacent organs can occur in the setting of widespread or necrotic disease and has been reported in only one case.^3^ No cases have been reported in association with Lynch syndrome.

Our patient underwent curative resection and received systemic therapy, and the latest imaging showed a complete response to treatment. However, 3 years after diagnosis, he developed this complication. This highlights the unpredictable aggressive nature of CRC, particularly in Lynch syndrome. Treatment of gastrohepatic fistulas depends on multiple factors like the cause, symptoms, or the etiology burden, and patient condition status. While surgical resection may be reasonable and curative in benign causes or localized malignancies, it is often not applicable in disseminated metastatic disease, as seen in our patient. The hepatic metastases demonstrated multisegment involvement, portal vein, and biliary invasion. Thus, a conservative palliative approach, including TPN, transfusion support, and systemic chemotherapy (FOLFIRI), was the most appropriate.

The genetic predisposition of Lynch syndrome is associated with an increased risk of urothelial carcinomas,^12^ and in our patient, a second primary urothelial carcinoma of the bladder was diagnosed during surveillance. Patients with Lynch syndrome and carriers require lifelong surveillance and interprofessional team follow-up.^13^

## Conclusion

This rare presentation of upper GI bleeding in a metastatic colorectal adenocarcinoma forming a gastrohepatic fistula in a patient with Lynch syndrome, with another primary urothelial carcinoma, outlines the aggressive behavior of CRC in a genetically predisposed patient and emphasizes the importance of long-term surveillance. A high index of suspicion for fistulizing disease should be maintained. The management of gastrohepatic fistula depends on multiple factors.
